# Organ size in small infants (The OSSI Study): establishing sonographic reference intervals for abdominal organs in preterm infants

**DOI:** 10.1007/s00431-026-07120-0

**Published:** 2026-05-28

**Authors:** Alexandros Rahn, Sabine Pirr, Corinna Peter, Carolin Böhne, Lara Klischke, Rieke Ringlstetter, Leonie Theis, Lars Brodowski, Bettina Bohnhorst, Doris Franke, Thomas Müller

**Affiliations:** 1 Department of Pediatric Pulmonology, Allergology and Neonatology, Hannover Medical School, Carl-Neuberg-Str. 1, 30625 Hannover, Germany; 2 Institut for Biometrics, Hannover Medical School, Carl-Neuberg-Str. 1, 30625 Hannover, Germany; 3 Department of Obstetrics and Gynecology, Hannover Medical School, Carl-Neuberg-Str. 1, 30625 Hannover, Germany; 4 Department of Pediatric Kidney, Liver and Metabolic Diseases, Hannover Medical School, Carl-Neuberg-Str. 1, 30625 Hannover, Germany

**Keywords:** Neonatal ultrasound, Preterm infants, Abdominal organs, Normative values, Reference intervals

## Abstract

Accurate ultrasound assessment of abdominal organ size in preterm infants is essential for evaluating growth and detecting abnormalities. However, standardized reference intervals for liver, spleen, and kidney dimensions in preterms below 40 cm body length are lacking. This study aimed to establish reference intervals for these organs. 57 eutrophic preterm infants with a body length below 40 cm (gestational age: 23 ^4^/_7_—31 ^5^/_7_ weeks; birth length: 29—39.5 cm) were prospectively examined using a standardized ultrasound protocol recommended by the German Society for Ultrasound in Medicine (DEGUM). Craniocaudal liver length, spleen length, and renal volume were measured and correlated with corrected gestational age, body weight, and body length. Reference intervals were calculated from log-transformed data with 95% confidence intervals for reference limits. Portal vein peak velocity before feeding and presence of a patent ductus venosus (PDV) were additionally assessed. Organ dimensions correlated most closely with body weight, followed by body length and corrected gestational age, with renal volume showing the highest correlations overall. Reference intervals for liver, spleen, and renal size showed tight 95% confidence intervals. Portal vein peak velocity ranged from 9—29 cm/s. A PDV was present in 56%; these infants were younger at examination (10.9 vs. 17 days, *p* = 0.01).

*Conclusion*: This study provides clinically relevant ultrasound reference intervals for abdominal organs in small preterm infants. These measurements may help distinguish physiological from pathological findings and guide clinical decision-making in neonatal care.

**Table:** 

**What is Known:**
• *Ultrasound is the preferred imaging modality for abdominal organ assessment in preterm infants, but standardized sonographic reference intervals for infants with a body length below 40 cm are lacking.*
**What is New:**
• *This study provides the first standardized sonographic reference intervals for liver length, splenic length, and renal volume in preterm infants below 40 cm body length, using a prospective, reproducible protocol aligned with DEGUM recommendations.*
• *Organ size correlated most strongly with body weight, followed by body length and corrected gestational age, with renal volume showing the highest overall correlations across all parameters.*

**Supplementary Information:**

The online version contains supplementary material available at 10.1007/s00431-026-07120-0.

## Introduction

Ultrasound (US) is the preferred imaging modality for evaluating abdominal organs in preterm infants, offering bedside availability, real-time imaging, and no exposure to ionizing radiation [1]. Beyond structural assessment, precise measurement of organ size is crucial, as enlargement of the liver, spleen, or kidneys can be associated with conditions like infection, congestive heart failure, metabolic disease, renal dysplasia, duplex kidney, renal vein thrombosis, or portal hypertension [2–8]. Because clinical assessment of organ size is often imprecise in preterm infants [9], US measurements are essential for guiding decisions and tracking growth.

Despite its clinical relevance, normative sonographic data for preterm infants with a body length below 40 cm remain scarce. Although several studies have reported US-based reference intervals for abdominal organs in preterm neonates [9–11], most focused on individual organs or employed measurement techniques that differ from those recommended by the German Society for Ultrasound in Medicine (DEGUM) and current protocols in pediatric US [12, 13], which reflect widely accepted international standards.

The aim of this study was to establish US reference intervals for craniocaudal liver size (in three standardized sagittal planes), splenic length, and renal volume in preterm infants with a body length below 40 cm, using a standardized approach aligned with international pediatric ultrasound practice to enable consistent evaluation and improve comparability across settings.

## Material and methods

### Study design and population

This prospective observational study included preterm infants with a body length below 40 cm at their first US examination. Participants were recruited from April 2024 to July 2025 in the level III neonatal intensive care unit (NICU) of a tertiary care center. The center manages about 2.500—3.000 births annually, including ~ 80—100 very low birth weight infants (< 1500 g). Eutrophic infants without conditions affecting abdominal organ size and a body length below 40 cm were included. Body length was measured supine using a flexible tape; weight undressed on a calibrated scale. Exclusion criteria were hypotrophy (birth weight < 10th percentile), hypertrophy (birth weight > 90th percentile), severe systemic infection, hepatopathy, major abdominal malformations (e.g., gastroschisis, omphalocele, diaphragmatic hernia), severe congenital heart defects, early death before imaging, and lack of consent. These clinical conditions were excluded as they may influence organ size. Severe systemic infection was defined as culture-proven sepsis or clinically diagnosed sepsis with laboratory evidence of systemic inflammation requiring systemic intravenous antibiotics. Hepatopathy included cholestasis (direct/conjugated bilirubin > 1 mg/dL, when available), elevated transaminases above age-specific reference ranges (when available), structural or vascular hepatic abnormalities on US, or known metabolic diseases affecting the liver including relevant newborn screening findings. In minor variants (e.g., polysplenia or duplex kidney), only the affected organ measurement was excluded; other values were kept. The protocol was approved by the local ethics committee (No. 11351_BO_K_2024). Written informed consent was obtained from the legal guardians of all participants prior to the examination. The principles of the Declaration of Helsinki were taken into account.

### Ultrasound protocol and organ measurements

All US examinations were performed using a GE Venue Go™ R4 US scanner (GE Healthcare Technologies, Chicago, IL, USA) with an 8 C micro-convex transducer to allow complete organ visualization across the entire body length range (30—39.5 cm) and to avoid probe-dependent measurement variability. Infants were examined supine before feeding without waking or sedation.

**Fig. 1:**
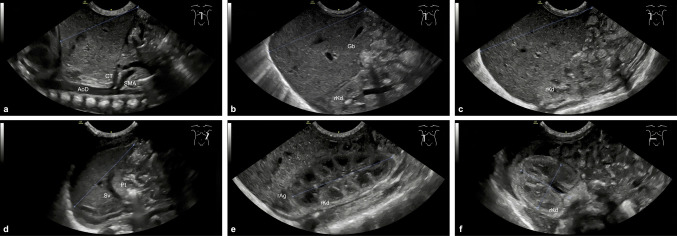
Standardized ultrasound planes for abdominal organ measurement. **a**: liver length measured in the midsternal line. **b**: liver length measured in the midclavicular line. **c**: liver length measured in the anterior axillary line. **d**: splenic length measured in a longitudinal oblique scan. **e**: right kidney imaged longitudinally from a frontal approach. **f**: right kidney imaged in transverse plane from a frontal approach. AoD: descending aorta, CT: coeliac trunk, Gb: gallbladder, Pt: pancreatic tail, rAg: right adrenal gland, rKd: right kidney, SMA: superior mesenteric artery, Sv: splenic vein

Two DEGUM level II pediatric examiners (6 and 9.5 years’ experience) used identical equipment, presets, and positioning. Interobserver reliability was previously assessed in a separate prospective study by our group [14], demonstrating excellent agreement with intraclass correlation coefficients ranging from 0.929—0.964 and Pearson correlation coefficients ranging from 0.930—0.965; Bland–Altman analysis additionally supported strong concordance between examiners. Craniocaudal liver length was measured in three defined sagittal planes to enhance measurement robustness and account for anatomical variability: along the midsternal line (MSL) using the aorta as a landmark, in the midclavicular line (MCL), typically at the level of the gallbladder, acknowledging its variable anatomical position, and in the anterior axillary line (AAL) at the longest craniocaudal alignment (see Fig. 1a-c). Splenic length was assessed in a left-sided oblique longitudinal scan including the hilum, measured from the upper to lower pole (see Fig. 1d). Renal volume was calculated using the ellipsoid formula: volume = length × width × depth × 0.523, with all dimensions obtained from a frontal approach (see Fig. 1e + f). Portal vein peak velocity at the liver hilum was measured by pulsed-wave Doppler in a hepatopetal direction with ≤ 60° angle correction. The presence of a patent ductus venosus (PDV) with detectable flow was also assessed. Corrected gestational age, weight, length, and head circumference were recorded at examination.

### Statistical analysis

All statistical analyses were performed using SAS 9.4 (SAS Institute Inc., Cary, NC, USA) and GraphPad Prism 10.4.1 (GraphPad Software, Boston, MA, USA). Normality was assessed by Shapiro–Wilk. test. Variables are described descriptively; normally distributed data as mean ± standard deviation (SD), non-normally distributed data as median and interquartile range (IQR).

As the primary objective of this study was the estimation of reference intervals rather than hypothesis testing, formal power calculations were not applicable. Given that reference intervals are intended to support future clinical decision-making, the uncertainty associated with their estimation should be minimized. As shown by Altman [15], sample sizes < 50 individuals may result in wide confidence intervals around the estimated reference limits, thereby reducing the precision. Accordingly, a minimum target sample size exceeding 50 preterm infants was defined,considering methodological recommendations and recruitment feasibility within the predefined study period in this narrowly defined population. Due to sample size and skewed distribution of some variables, logarithmic transformation was applied to all liver, spleen, and kidney variables prior to reference interval calculation. Histograms of original and transformed data are provided in Supplemental Fig. 1.

Reference intervals were calculated on the log scale using mean ± 1.96 × SD. To improve interpretability, log-transformed data were back-transformed to the original scale. Additionally, 95% confidence intervals (CIs) for the lower and upper limits were calculated to quantify precision [15].

Further analyses included group comparisons using parametric (t-test) or non-parametric methods (Mann–Whitney U test; MWU), as appropriate, as well as correlations between organ measurements and clinical parameters (corrected gestational age, body weight, and body length at the time of examination) using Pearson’s correlation coefficient (*r*). Correlation analyses were performed for exploratory and descriptive purposes. The two-sided type-I error rate was set to α = 0.05, with statistical significance defined as *p* < 0.05.

## Results

**Fig. 2:**
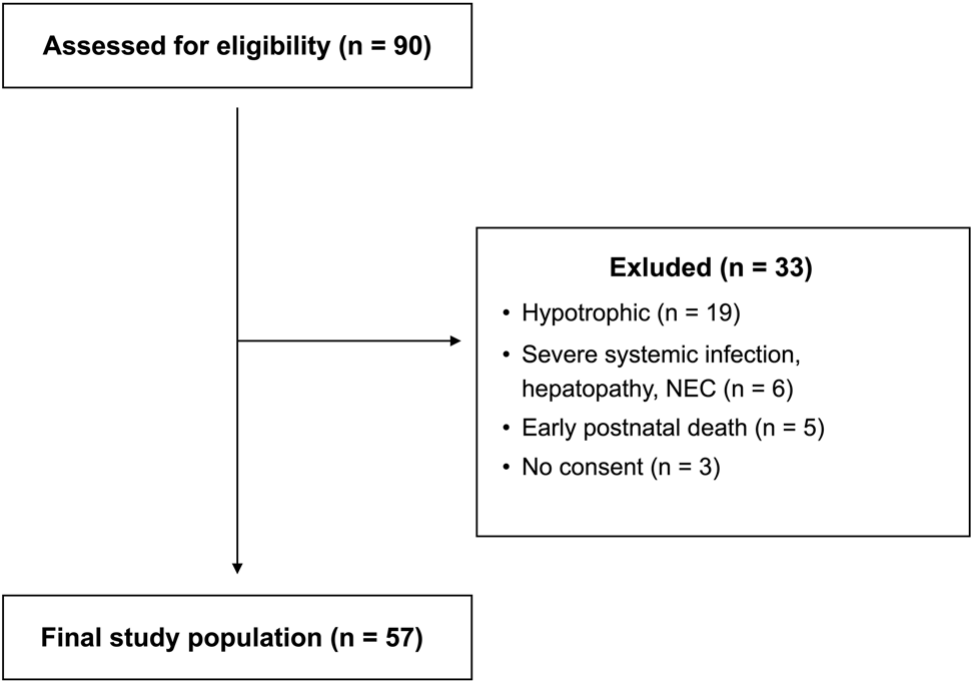
Participant flow diagram: overview of screening, exclusions, and final inclusion for abdominal organ measurements

A total of 90 preterm infants were screened, of whom 57 met inclusion criteria and were analyzed (see Fig. 2). The final cohort included 29 females and 28 males. Mean gestational age at birth was 27 ^1^/_7_ ± 2 weeks, with birth weight ranging from 500 to 1450 g and a median birth length of 36 cm (range: 29 cm—39.5 cm). The first US examination was performed at a median age of 14 days (range: 2—32), with no sex difference in body length (*p* = 0.562, MWU). Liver measurements were complete; renal volume was missing in one infant (duplex kidney) and spleen length in another (polysplenia), yielding 56 datasets for both. No sex differences were observed for any organ measurements (t-test; *p* = 0.23—0.94, see Supplemental Table 1). A PDV was present in 56% of infants, who were younger at examination than those without PDV (median 10.9 vs. 17 days, *p* = 0.014, MWU). Further details, including organ sizes and flow measurements, are provided in Table 1.

**Table 1:** Demographic, clinical, and sonographic characteristics of the study population

Characteristics	Value^*^
Number of infants	57
Sex	Female: 29 (50.9%) Male: 28 (49.1%)
Gestational age (p.m., weeks)	27 ^1^/_7_ ± 2 Range: 23 ^4^/_7_—31 ^5^/_7_
Birth weight (g)	970 ± 260 Range: 500—1450
Birth weight percentile	40.44 ± 18.90 Range: 10—90
Birth length (cm)	36 (33—38) Range: 29—39.5
Birth length percentile	40 (30—60) Range: 1—90
Birth head circumference (cm)	25.17 ± 2.26 Range: 20—29
Birth head circumference percentile	42.25 ± 19.76 Range: 6—90
Body surface area (Mosteller, m^2^)	0.1 (0.08—0.11) Range: 0.06—0.13
Age at first examination (days)	14 (6—16) Range: 2—32
Weight at first examination (g)	1020 ± 250 Range: 560—1600
Length at first examination (cm)	36 (35—39) Range: 30—39.5
Liver length in MSL (cm)	3.22 ± 0.46 Range: 2.1—4.1
Liver length in MCL (cm)	3.87 ± 0.44 Range: 3.0—4.8
Liver length in AAL (cm)	3.89 ± 0.52 Range: 2.7—5.7
Spleen length (cm), *n* = 56	2.70 ± 0.36 Range: 1.9—3.4
Right kidney volume (ml), *n* = 56	6 (4—7) Range: 2—10
Left kidney volume (ml), *n* = 56	5 (4—6) Range: 2—9
Portal vein peak velocity (cm/s), *n* = 53	17.42 ± 4.37 Range: 9—29
Patent ductus venosus (*n*)	32 (56%)
AST at examination (± 7 days; U/l), *n* = 34	29.24 ± 7.29 Range: 16—52
ALT at examination (± 7 days; U/l), *n* = 33	7 (4—10) Range: 4—16
Creatinine at examination (± 7 days; µmol/l), *n* = 52	55.20 ± 13.40 Range: 20—77

^*^ = Non-normally distributed data are shown as median (interquartile range), whereas normally distributed data are presented as mean ± SD. Kidney volume and spleen length were assessed in 56 infants, as one infant with a duplex kidney and another with polysplenia were excluded from these respective measurements. *AAL* anterior axillary line, *ALT* alanine aminotransferase, *AST* aspartate aminotransferase, *MCL* midclavicular line, *MSL* midsternal line

Mean values, SD, and the 95% reference intervals with corresponding 95% CIs for the lower and upper boundaries are summarized in Table 2. The reference intervals, derived from log-transformed data, describe the estimated organ size ranges for liver, spleen, and kidneys in preterm infants with a body length below 40 cm.

**Table 2:** Sonographic reference intervals with 95% confidence intervals of the corresponding reference interval boundaries for abdominal organ size in preterm infants below 40 cm body length

Characteristics	N	Mean (SD), log scale	95% RI*	95% CI of the lower limit of the RI	95% CI of the upper limit of the RI
Liver length in MSL (cm)	57	1.16 (0.15)	2.38—4.27	2.23—2.55	3.99—4.56
Liver length in MCL (cm)	57	1.35 (0.12)	3.07—4.82	2.90—3.24	4.55—5.07
Liver length in AAL (cm)	57	1.35 (0.13)	2.99—5.00	2.81—3.15	4.72—5.30
Spleen length (cm)	56	0.98 (0.14)	2.04—3.51	1.91—2.17	3.31—3.76
Right kidney volume (ml)	56	1.69 (0.34)	2.78—10.49	2.38—3.24	8.99—12.23
Left kidney volume (ml)	56	1.60 (0.34)	2.56—9.56	2.19—2.99	8.21—11.18

*Reference intervals and confidence intervals were derived using log-transformed data that were then back-transformed for better interpretability. *AAL* anterior axillary line, *CI* confidence interval, *MCL* midclavicular line, *MSL* midsternal line, *RI* reference interval, *SD* standard deviation

**Fig. 3:**
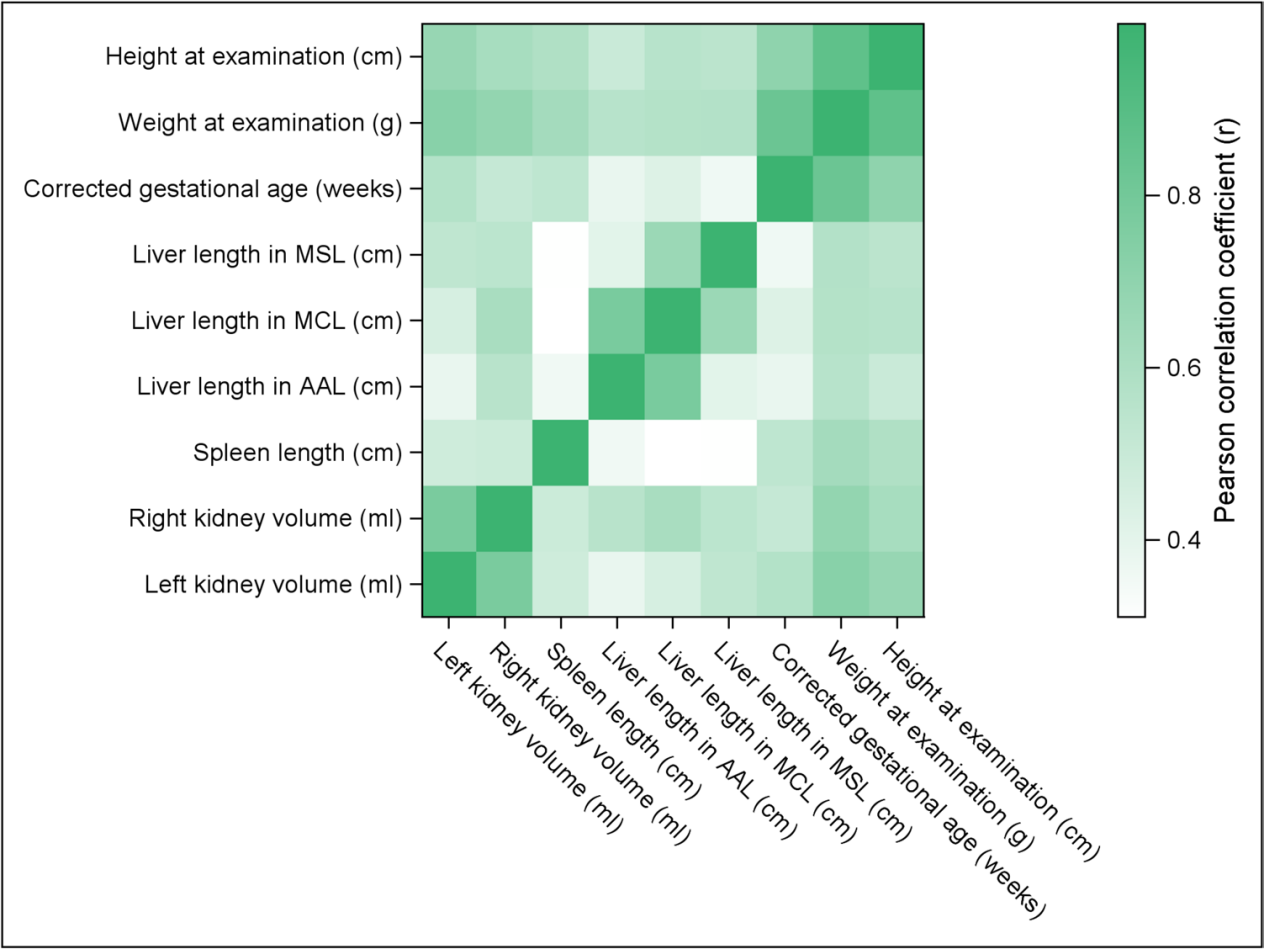
Heatmap of pairwise associations between organ measurements and clinical parameters using the Pearson correlation coefficient (r). Darker colors indicate stronger positive correlations. AAL: anterior axillary line, MCL: midclavicular line, MSL: midsternal line

Organ size correlated strongest with body weight (r = 0.56—0.73, moderate—strong), followed by body length (r = 0.50—0.68, moderate—strong) and corrected gestational age (r = 0.36—0.59, weak—moderate). Overall, correlations were highest for renal volume, followed by spleen and liver length (see Fig. 3 and Supplemental Table 2). Percentile plots are shown in Supplemental Fig. 2.

## Discussion

To our knowledge, this is the first study providing standardized US reference values for liver length, spleen length, and kidney volume in preterm infants with a body length below 40 cm, based on a structured protocol aligned with DEGUM recommendations.

The clinical applicability of these reference intervals rests on their role as a structured framework for identifying organ size deviations that warrant further evaluation, particularly given that palpation is inherently imprecise in preterm infants [9]. Accurate reference intervals are therefore essential in neonatal US, as hepatomegaly, splenomegaly, or renal enlargement may indicate serious underlying pathology and may go unrecognized without normative comparisons. Measurements exceeding the reference limit should prompt systematic evaluation, as hepatomegaly may indicate conditions such as congestive heart failure, sepsis, or metabolic liver disease [2, 5]; splenomegaly infection or portal hypertension [7]; and renal size abnormalities renal vein thrombosis or dysplasia [4, 8].

Reference intervals are equally essential for longitudinal monitoring, and size-adjusted charts improve diagnostic accuracy by distinguishing physiological variation from true pathology [16]. Validated outcome-based thresholds for abdominal organ size in this preterm population do not currently exist in the literature; establishing such thresholds requires prospective outcome data, which is beyond the scope of this normative study. The present dataset provides the necessary foundation upon which such analyses can subsequently be built.

Despite their clinical importance, no standardized reference intervals exist for infants below 40 cm in body length. This study addresses this gap by providing organ-specific reference intervals using a reproducible protocol consistent with DEGUM and current pediatric US standards [12, 13]. The dataset includes 95% CIs for the reference limits and percentile plots relating organ size to body length, weight, and corrected gestational age, offering a practical framework for clinical assessment.

Previous studies reported abdominal organ measurements in term and preterm infants but differ in protocols, cohort definitions, and measurement techniques, limiting comparability with our findings. Soyupak et al. measured liver length in the MCL and reported kidney and spleen dimensions in neonates between 24 and 41 weeks of gestation, but data were not stratified by body length, and results for smaller preterm infants were not presented separately [10]. Megremis et al. examined splenic length in preterm neonates over the first three postnatal months, using maximal length in a longitudinal coronal view. Their study provided postnatal percentile curves but differs in scan approach and study design, limiting direct comparison [11]. Kahramaner et al. reported liver and spleen lengths in preterm neonates grouped by different body length intervals. Although the scan orientation was similar, the cohort was grouped differently and not restricted to a clearly defined body length range as in our study, which limits direct comparability. Additionally, kidney measurements were not included [9]. In summary, while these studies provide relevant normative data, differences in measurement technique, cohort grouping, and organs assessed must be considered. The present study complements existing data by providing consistent reference intervals for liver, spleen, and kidney size in preterm infants below 40 cm, based on a standardized, reproducible US protocol. While this study focuses on morphometric reference intervals, functional evaluation of abdominal organs remains clinically important. Infants were clinically assessed at the time of examination, and available laboratory parameters obtained close to imaging (transaminases, creatinine) did not indicate organ dysfunction. Sonographic, laboratory, and clinical findings should therefore be interpreted in a complementary manner in clinical practice.

Organ dimensions in our cohort showed moderate to strong associations with body weight and body length, whereas correlations with gestational age were weaker. As these analyses were exploratory, they characterize associations rather than independent or causal effects. The strongest associations were observed for renal volume, which may be related to several biological and methodological factors: anatomically, the kidneys grow in close proportion to overall body size, and renal development continues into late gestation, making kidney size a sensitive maturational marker in preterm infants [17]. In addition, renal volume was assessed using a three-dimensional approach (length × width × depth × 0.523), which may enhance measurement accuracy. Unlike single linear measures, volumetric estimation captures growth in all spatial dimensions and can reflect subtle morphological differences not represented by length alone. This more comprehensive anatomical representation achieved through volumetric measurement may explain the stronger correlation observed for renal volume compared to single-axis measures of the other organs. Although weight showed the strongest correlation with organ size, body length was selected as the reference parameter to ensure consistency across organs and to align with existing normative values, particularly for liver and spleen measurements. For comparability, the same approach was used for renal volume.

Previous studies have reported inconsistent findings regarding the most suitable anthropometric reference parameter, with some identifying body length [16, 18, 19] and others body weight [10, 20] as the stronger correlate of abdominal organ size in neonates. In our dataset, body length showed a meaningful correlation and, unlike weight, provides a stable anatomical reference not influenced by short-term fluid shifts. While length measurements in neonates can be operator-dependent, especially with flexible tape measures rather than rigid boards, board-based techniques are often not feasible in preterm infants, in whom minimal handling is essential [21, 22]. In our study, body length was measured supine using a standardized tape-based approach, reflecting routine practice. Gestational age correlated least with organ dimensions, likely due to biological variability in fetal growth and limitations of dating methods, which rely on last menstrual period or early US and have known error margins [23].

While the primary focus was on organ size, we also assessed portal vein peak velocity and documented whether the ductus venosus Arantii was still patent or functionally closed. Because feeding alters splanchnic blood flow, all Doppler measurements were performed shortly before scheduled feedings to reduce variability.

The observed portal vein peak flow range (9—29 cm/s) may support clinical interpretation in preterm infants, particularly when hepatic perfusion abnormalities or circulatory instability are suspected. As a functional marker of splanchnic blood flow, portal vein velocity reflects the interplay of systemic hemodynamics, hepatic vascular architecture, and parenchymal resistance [24]. It can be influenced by feeding status, ductus arteriosus patency, and structural liver changes [25]. Although not routinely assessed in neonatal US, it may provide useful clinical context when hepatic dysfunction or impaired perfusion is suspected.

Given its role in fetal circulation, postnatal patency of the ductus venosus Arantii may affect early hepatic hemodynamics and should be considered when interpreting Doppler findings. It originates from the left portal vein and runs dorsocranially in the fissura ligamenti venosi to the inferior vena cava. A patent ductus venosus (PDV) was present in 56% of infants. Those with PDV were significantly younger at imaging than those with spontaneous closure, consistent with a time-dependent closure pattern: in term neonates, the ductus venosus typically closes within the first days of life, whereas in preterm infants patency may persist for weeks or longer [26]. Recognizing this physiologic variation is essential for interpreting early postnatal Doppler studies. A PDV should not be mistaken for a pathological shunt, as long as the vessel follows its typical anatomical course and the finding occurs within the expected temporal window. While generally benign, PDV may rarely be associated with hypoglycemia or neurocognitive symptoms due to portosystemic bypass [27].

### Strengths and limitations

A notable strength of this study is the focus on a well-defined and clinically relevant subgroup of preterm infants below 40 cm in body length. The study provides organ-specific reference intervals in a single dataset, together with 95% CIs for interval boundaries, allowing direct quantification of estimation precision. While the reference intervals define ranges covering 95% of the population, the 95% CIs quantify the precision of these limits. Notably, these CIs were overall narrow, which may reflect reasonable precision, though interpretation should consider the underlying sample size in this specific preterm cohort.

Importantly, 57 infants were examined within this small body length range of 30—39.5 cm alone. In contrast, available liver size reference data are based on only 90 children across a much broader range of 40—180 cm (14 subgroups), and spleen length references originate from 136 children between 35 and 169 cm (11 subgroups) [13].

All examinations were performed by two experienced pediatric sonographers with DEGUM level II certification, using uniform equipment and a predefined acquisition protocol. The addition of a midclavicular liver measurement, which is widely used as a primary single-plane approach in routine clinical practice worldwide but not explicitly required by DEGUM guidelines, enhances the transferability of the protocol. In contrast to this single-plane approach, the inclusion of two additional standardized sagittal planes provides complementary anatomical information and may improve the robustness of liver assessment.

Interobserver agreement was high for all measurements (Pearson and Intraclass correlation coefficients > 0.9), as shown in a separately published validation study [14]. The prospective design ensured predefined eligibility criteria, controlled examination conditions, and consistent data acquisition throughout the study period, thereby minimizing measurement heterogeneity and potential selection bias. Furthermore, the systematic analysis of correlations between organ size and somatic parameters (body weight and body length) as well as developmental factors (gestational age) supports interpretation and clinical applicability.

Nonetheless, several limitations must be acknowledged. The study was conducted at a single tertiary care center, which may limit generalizability in settings with different clinical routines, US expertise, or patient characteristics. Preterm infants constitute a heterogeneous population; however, we aimed to reduce variability by applying strict inclusion and exclusion criteria and by restricting the cohort to eutrophic infants without major comorbidities. Body weight showed the strongest correlation with organ size, yet reference intervals were primarily stratified by body length to align with existing normative datasets and to avoid excessive subgrouping, which could have reduced statistical robustness. Potential differences in organ growth trajectories across the gestational age range studied cannot be fully excluded; however, corrected gestational age showed the weakest correlation with organ size across all parameters. Graphical distributions by corrected gestational age are additionally provided in Supplemental Fig. 2. The sample sizes (*n* = 56 for spleen length and renal volume, and *n* = 57 for liver length) were modest and may have affected how finely the reference limits could be estimated. Lastly, the cross-sectional study design does not capture longitudinal changes in organ size over time. This study intentionally focused on infants below 40 cm body length, as reference data for larger children are already available [13]; longitudinal assessment of organ growth trajectories across the broader preterm period represents a valuable avenue for future research.

## Conclusion

This study provides standardized reference intervals for liver length, splenic length, and renal volume in preterm infants with a body length below 40 cm, using a consistent and clinically applicable US protocol. These values support structured assessment of abdominal organ size in early neonatal care and provide a framework for identifying deviations that may warrant further clinical evaluation. The present dataset constitutes a necessary normative foundation upon which future diagnostic thresholds and outcome-based studies can be built. External multicenter validation will be important before broad clinical adoption. Beyond cross-sectional normative data, longitudinal assessment of organ growth trajectories (including organ-specific growth curves and z-score models based on serial measurements in individual infants) represents an important next step and a distinct scientific question that prospective studies should address.

## Supplementary Information

Below is the link to the electronic supplementary material.

Supplementary Figure 1 (PDF 1.57 MB)

Supplementary Figure 2 (PDF 2.19 MB)

Supplementary Table 1 (PDF 103 KB)

Supplementary Table 2 (PDF 86.1 KB)

## Data Availability

The datasets generated and analyzed during this study are not publicly available due to patient confidentiality regulations but are available from the corresponding author upon reasonable request.
