# Supplementary material for: Organ size in small infants (The OSSI Study): establishing sonographic reference intervals for abdominal organs in preterm infants
Source: Eur J Pediatr. 2026 May 28;185(6):446. doi: 10.1007/s00431-026-07120-0 (PMC13219109; doi:10.1007/s00431-026-07120-0)
Supplement: Supplementary file 1 — Supplementary Figure 1 (PDF 1.57 MB) [file 431_2026_7120_MOESM1_ESM.pdf]

Supplemental Figure 1 Histograms of liver length (MSL, MCL, AAL), spleen length, and renal volumes before (left) and after log-transformation (right), illustrating the reduction of skewness following transformation.

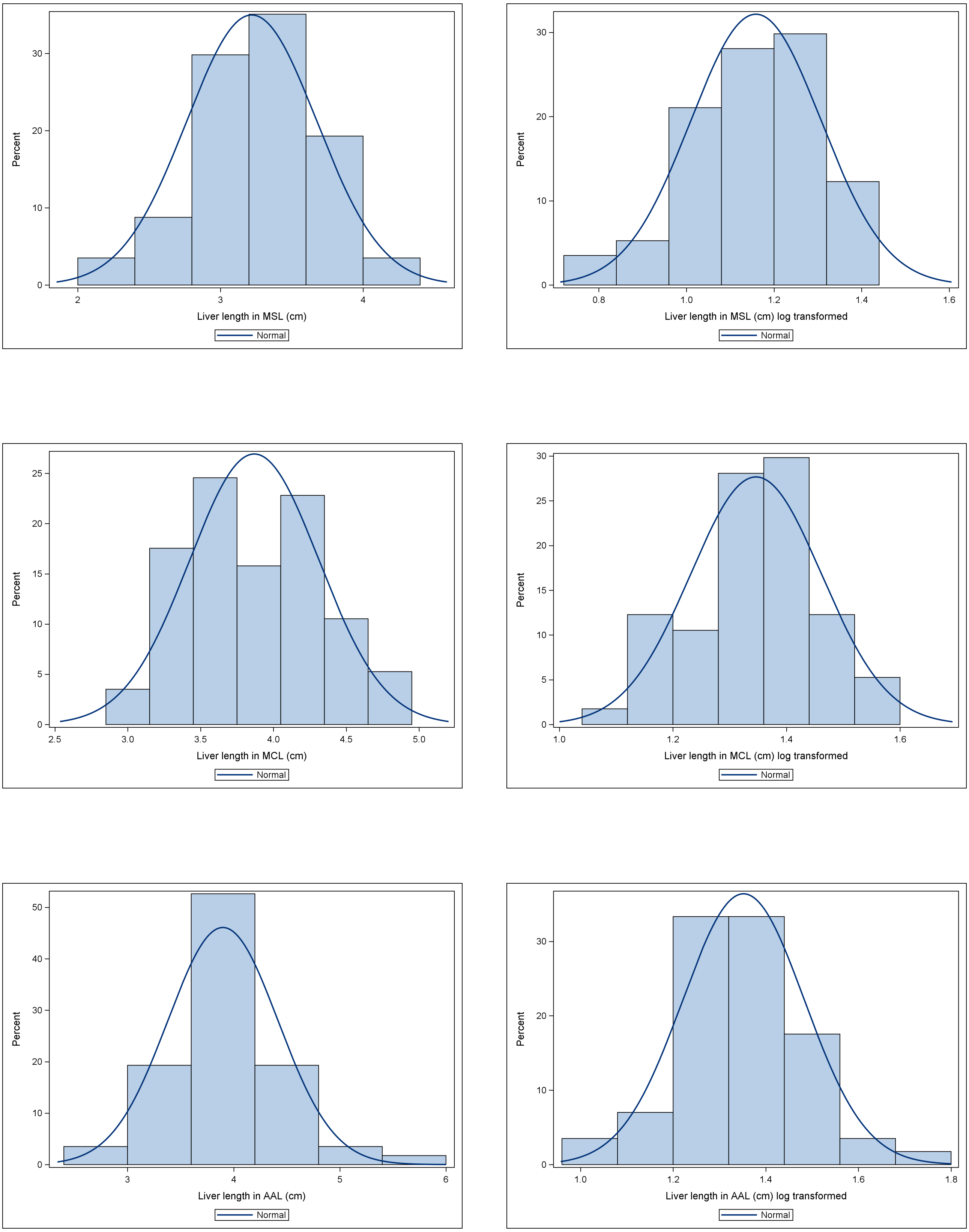

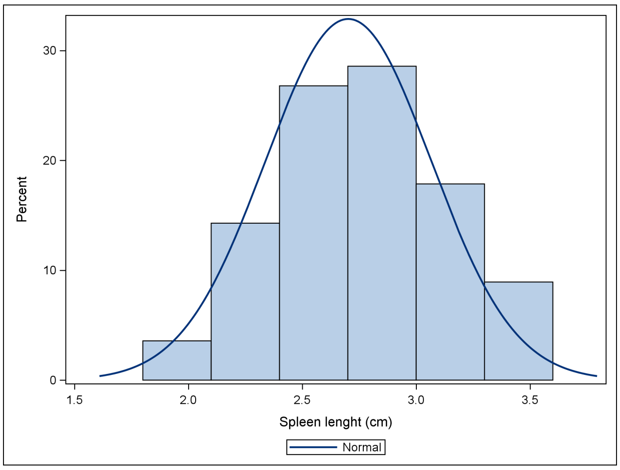

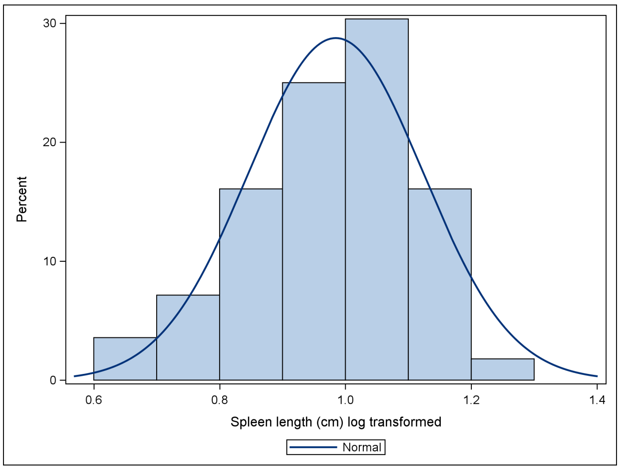

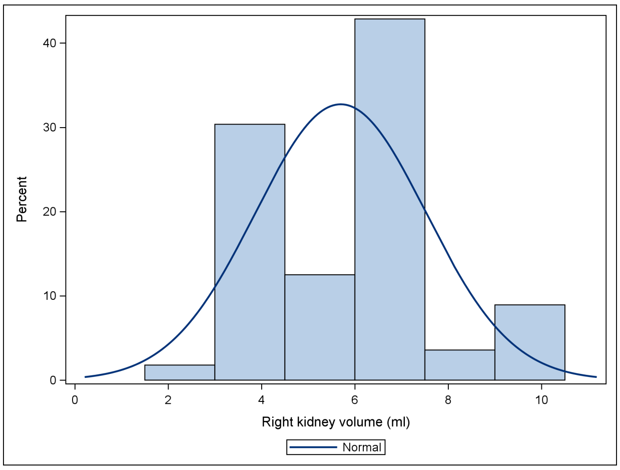

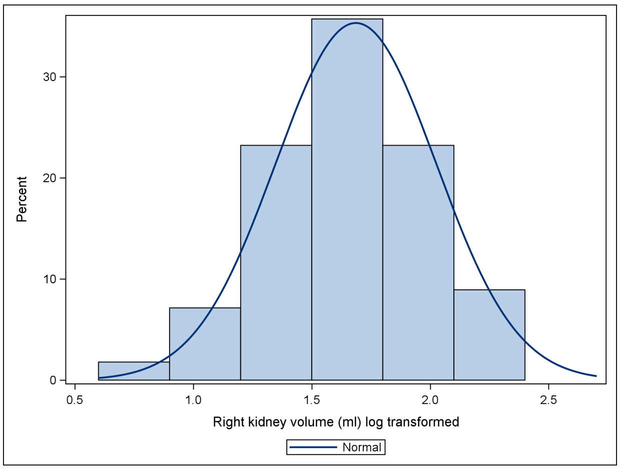

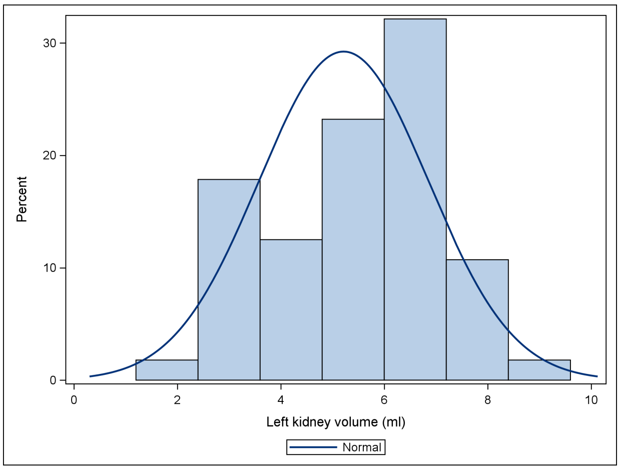

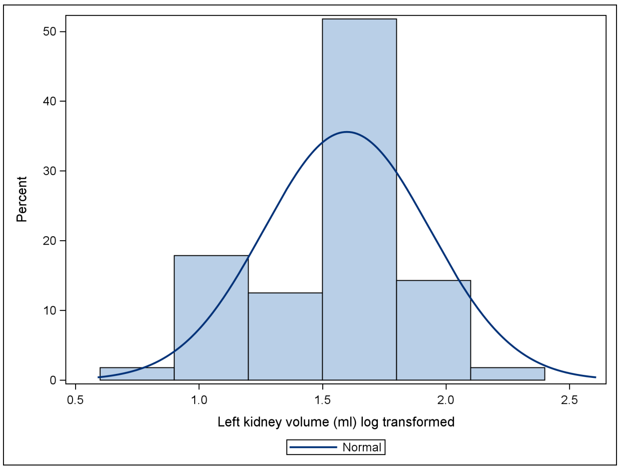

AAL: anterior axillary line, MCL: midclavicular line, MSL: midsternal line.
