# Supplementary material for: Organ size in small infants (The OSSI Study): establishing sonographic reference intervals for abdominal organs in preterm infants
Source: Eur J Pediatr. 2026 May 28;185(6):446. doi: 10.1007/s00431-026-07120-0 (PMC13219109; doi:10.1007/s00431-026-07120-0)
Supplement: Supplementary file 2 — Supplementary Figure 2 (PDF 2.19 MB) [file 431_2026_7120_MOESM2_ESM.pdf]

Supplemental Figure 2 Percentile plots of liver length (MSL, MCL, AAL), spleen length, and renal volumes in relation to body weight, body length and gestational age at the time of examination.

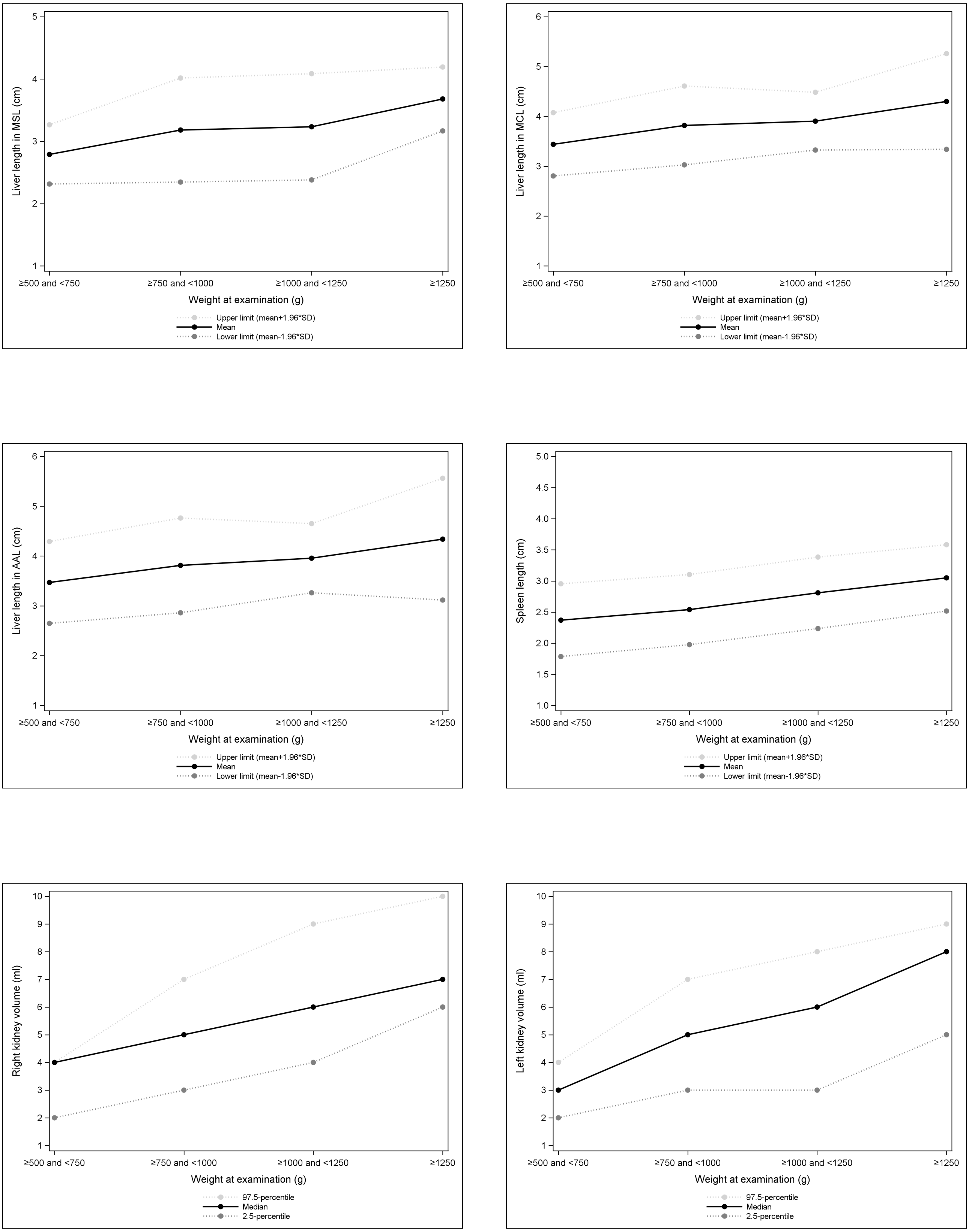

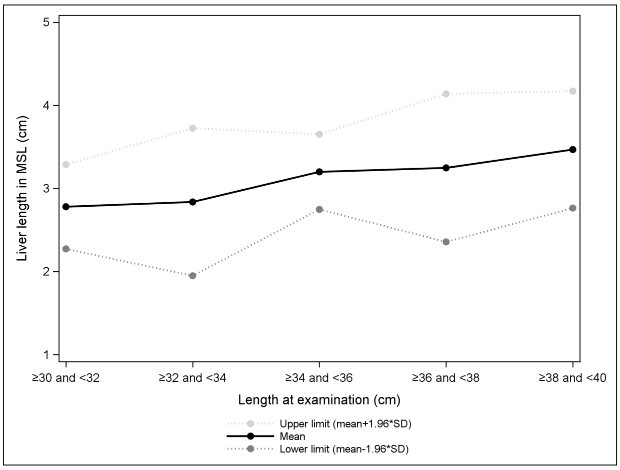
Liver length in MSL (cm)
Length at examination (cm)
≥30 and <32
≥32 and <34
≥34 and <36
≥36 and <38
≥38 and <40
Upper limit (mean+1.96*SD)
Mean
Lower limit (mean-1.96*SD)

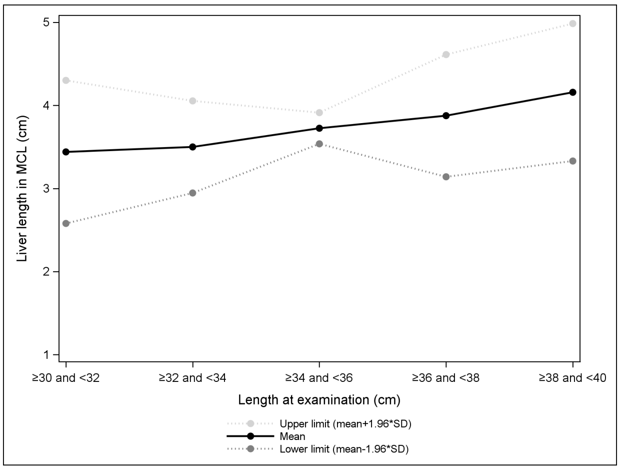
Liver length in MCL (cm)
Length at examination (cm)
≥30 and <32
≥32 and <34
≥34 and <36
≥36 and <38
≥38 and <40
Upper limit (mean+1.96*SD)
Mean
Lower limit (mean-1.96*SD)

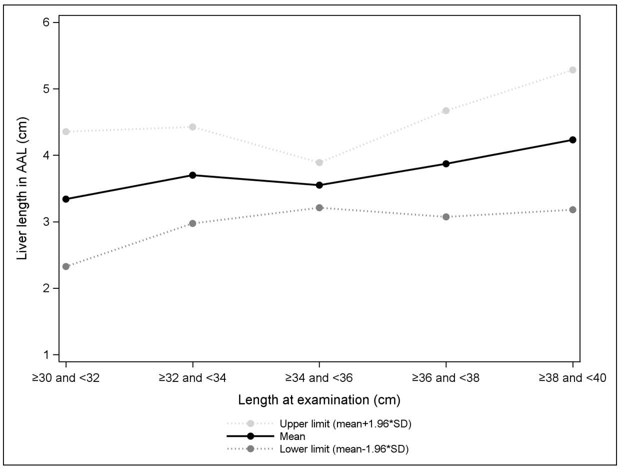
Liver length in AAL (cm)
Length at examination (cm)
≥30 and <32
≥32 and <34
≥34 and <36
≥36 and <38
≥38 and <40
Upper limit (mean+1.96*SD)
Mean
Lower limit (mean-1.96*SD)

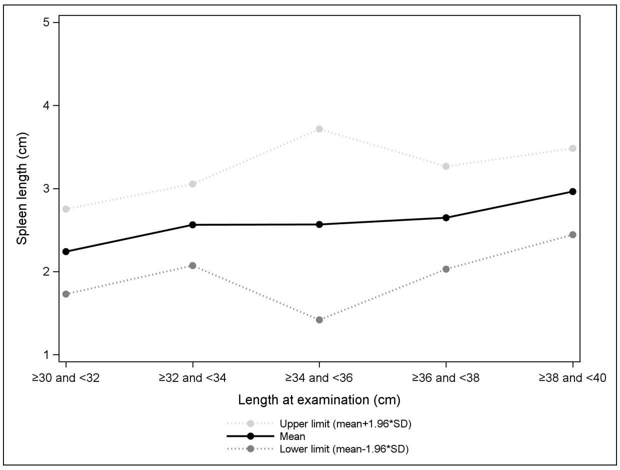
Spleen length (cm)
Length at examination (cm)
≥30 and <32
≥32 and <34
≥34 and <36
≥36 and <38
≥38 and <40
Upper limit (mean+1.96*SD)
Mean
Lower limit (mean-1.96*SD)

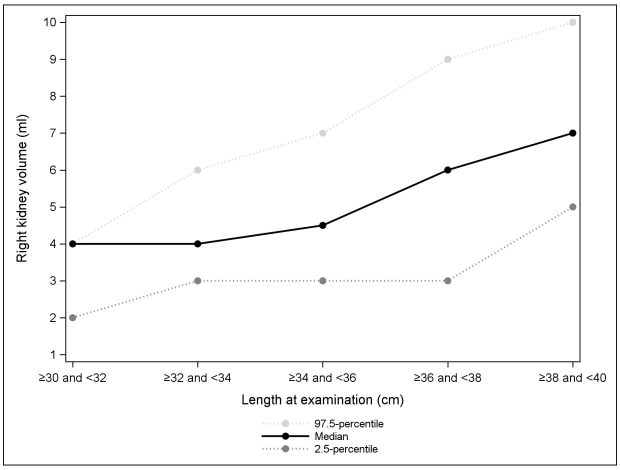
Right kidney volume (ml)
Length at examination (cm)
≥30 and <32
≥32 and <34
≥34 and <36
≥36 and <38
≥38 and <40
97.5-percentile
Median
2.5-percentile

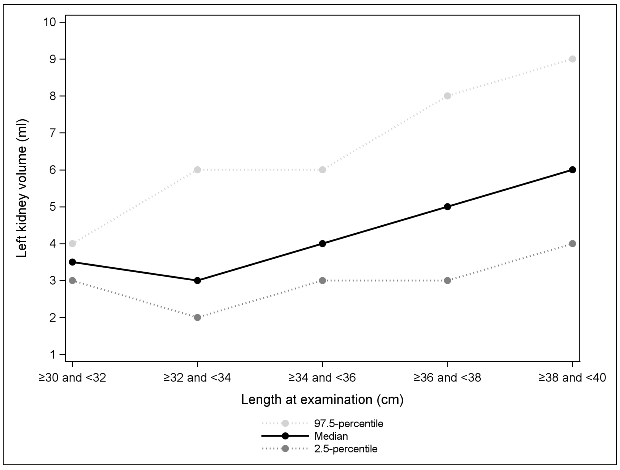
Left kidney volume (ml)
Length at examination (cm)
≥30 and <32
≥32 and <34
≥34 and <36
≥36 and <38
≥38 and <40
97.5-percentile
Median
2.5-percentile

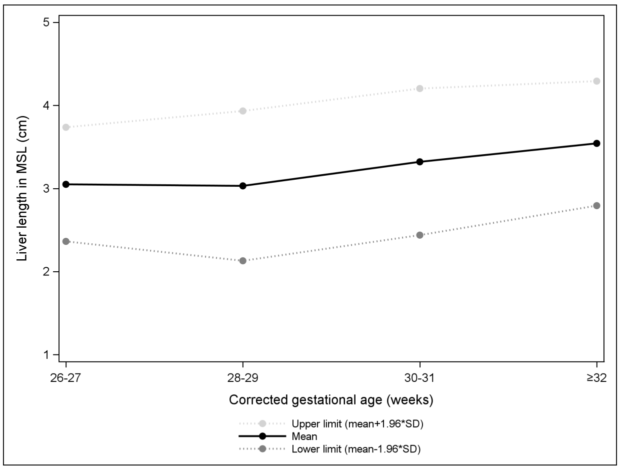

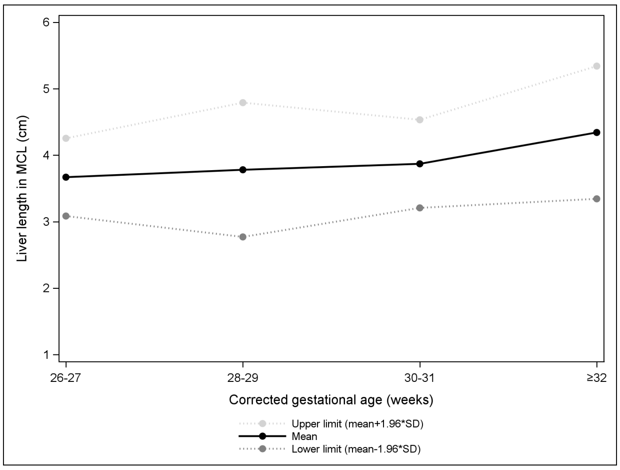

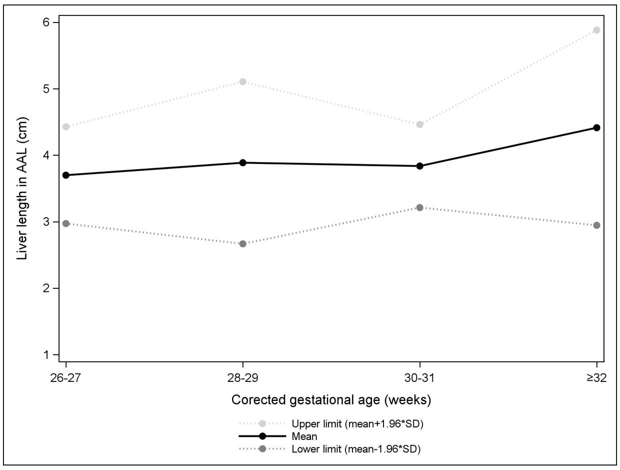

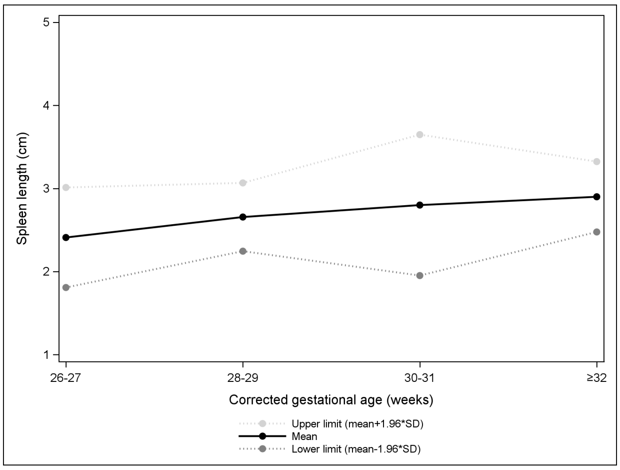

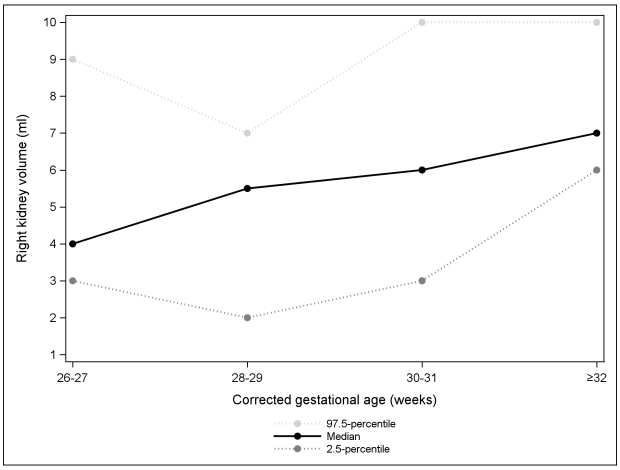

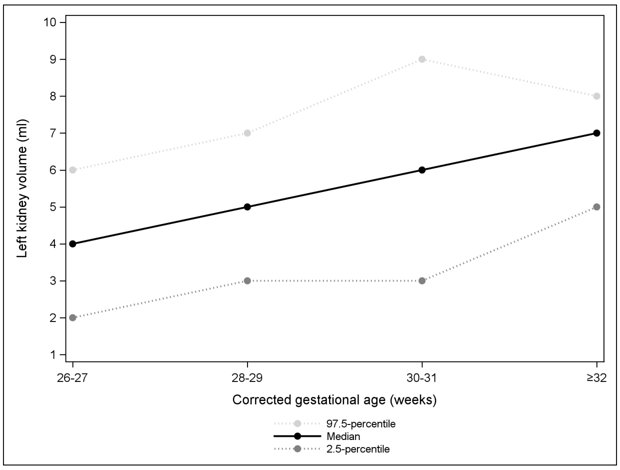

For normally distributed variables (liver length and spleen length), the lower and upper limits were derived as mean ± 1.96 × SD. For non-normally distributed variables (renal volumes), the median together with the empirical 2.5th and 97.5th percentiles was used. AAL: anterior axillary line, MCL: midclavicular line, MSL: midsternal line.
