# Supplementary material for: Organ size in small infants (The OSSI Study): establishing sonographic reference intervals for abdominal organs in preterm infants
Source: Eur J Pediatr. 2026 May 28;185(6):446. doi: 10.1007/s00431-026-07120-0 (PMC13219109; doi:10.1007/s00431-026-07120-0)
Supplement: Supplementary file 3 — Supplementary Table 1 (PDF 103 KB) [file 431_2026_7120_MOESM3_ESM.pdf]

Supplemental Table 1 Comparison of organ measurements between male and female preterm infants: liver length in MSL (a), MCL (b), and AAL (c), spleen length (d), right kidney volume (e), and left kidney volume (f). Data are presented as mean ± standard deviation (SD) and median (range), mean difference (male - female) with 95% confidence interval (CI), and corresponding p-values (t-test)*.

a) Liver length in MSL (cm)

	**Male, n = 28**	**Female, n = 29**	**Total, n = 57**
n (missing)	28 (0)	29 (0)	57 (0)
Mean (± SD)	3.24 (± 0.46)	3.2 (± 0.46)	3.22 (± 0.46)
Median (range)	3.3 (2.1 - 4.1)	3.1 (2.1 - 4)	3.2 (2.1 - 4.1)
Mean difference			0.04
95% CI mean difference			-0.2 - 0.28
P-value (t-test)			0.7483

b) Liver length in MCL (cm)

	**Male, n = 28**	**Female, n = 29**	**Total, n = 57**
n (missing)	28 (0)	29 (0)	57 (0)
Mean (± SD)	3.84 (± 0.40)	3.9 (± 0.49)	3.87 (± 0.44)
Median (range)	3.9 (3.2 - 4.5)	3.8 (3 - 4.8)	3.8 (3 - 4.8)
Mean difference			-0.06
95% CI mean difference			-0.29 - 0.18
P-value (t-test)			0.6311

c) Liver length in AAL (cm)

	**Male, n = 28**	**Female, n = 29**	**Total, n = 57**
n (missing)	28 (0)	29 (0)	57 (0)
Mean (± SD)	3.86 (± 0.43)	3.93 (± 0.60)	3.89 (± 0.52)
Median (range)	3.9 (3.1 - 4.8)	3.9 (2.7 - 5.7)	3.9 (2.7 - 5.7)
Mean difference			-0.07
95% CI mean difference			-0.34 - 0.21
P-value (t-test)			0.6320

d) Spleen length (cm)

	Male, n = 28	Female, n = 29	Total, n = 57
n (missing)	28 (0)	28 (1)	56 (1)
Mean (± SD)	2.76 (± 0.33)	2.64 (± 0.39)	2.70 (± 0.36)
Median (range)	2.8 (2.1 - 3.3)	2.7 (1.9 - 3.4)	2.7 (1.9 - 3.4)
Mean difference			0.12
95% CI mean difference			-0.08 - 0.31
P-value (t-test)			0.2291

e) Right kidney volume (ml)

	Male, n = 28	Female, n = 29	Total, n = 57
n (missing)	28 (0)	28 (1)	56 (1)
Mean (± SD)	5.71 (± 1.72)	5.68 (± 1.96)	5.70 (± 1.83)
Median (range)	6 (3 - 10)	6 (2 - 10)	6 (2 - 10)
Mean difference			0.03
95% CI mean difference			-0.95 - 1.02
P-value (t-test)			0.9425

f) Left kidney volume (ml)

	Male, n = 28	Female, n = 29	Total, n = 57
n (missing)	28 (0)	28 (1)	56 (1)
Mean (± SD)	5.14 (± 1.72)	5.29 (± 1.58)	5.21 (± 1.64)
Median (range)	5 (3 - 8)	5.5 (2 - 9)	5 (2 - 9)
Mean difference			-0.15
95% CI mean difference			-1.03 - 0.74
P-value (t-test)			0.7473

* For all parameters, additional Mann-Whitney U tests were performed; results were consistent and did not change statistical significance. Kidney volume and spleen length were assessed in 56 infants, as one infant with a duplex kidney and another with polysplenia were excluded from these respective measurements. AAL: anterior axillary line, MCL: midclavicular line, MSL: midsternal line.
