# Supplementary material for: Organ size in small infants (The OSSI Study): establishing sonographic reference intervals for abdominal organs in preterm infants
Source: Eur J Pediatr. 2026 May 28;185(6):446. doi: 10.1007/s00431-026-07120-0 (PMC13219109; doi:10.1007/s00431-026-07120-0)
Supplement: Supplementary file 4 — Supplementary Table 2 (PDF 86.1 KB) [file 431_2026_7120_MOESM4_ESM.pdf]

Supplemental Table 2 Pearson correlation coefficients (*r*), 95% confidence intervals and corresponding p-values for associations between organ measurements and body weight (a), body length (b), and corrected gestational age at examination (c)

a) Correlation with body weight at examination

Organ dimension	*r*	95% CI	P value
Liver length in MSL (cm)	0.58	0.38 - 0.73	<0.0001
Liver length in MCL (cm)	0.58	0.37 - 0.73	<0.0001
Liver length in AAL (cm)	0.56	0.36 - 0.72	<0.0001
Spleen length (cm)	0.63	0.44 - 0.77	<0.0001
Right kidney volume (ml)	0.70	0.53 - 0.81	<0.0001
Left kidney volume (ml)	0.73	0.58 - 0.83	<0.0001

b) Correlation with body length at examination

Organ dimension	*r*	95% CI	P value
Liver length in MSL (cm)	0.55	0.34 - 0.71	<0.0001
Liver length in MCL (cm)	0.57	0.36 - 0.72	<0.0001
Liver length in AAL (cm)	0.50	0.28 - 0.68	<0.0001
Spleen length (cm)	0.59	0.39 - 0.74	<0.0001
Right kidney volume (ml)	0.62	0.43 - 0.76	<0.0001
Left kidney volume (ml)	0.68	0.50 - 0.80	<0.0001

c) Correlation with corrected gestational age at examination

Organ dimension	*r*	95% CI	P value
Liver length in MSL (cm)	0.36	0.10 - 0.56	0.0068
Liver length in MCL (cm)	0.43	0.19 - 0.62	0.0008
Liver length in AAL (cm)	0.38	0.13 - 0.58	0.0037
Spleen length (cm)	0.53	0.31 - 0.70	<0.0001
Right kidney volume (ml)	0.52	0.30 - 0.69	<0.0001
Left kidney volume (ml)	0.59	0.39 - 0.74	<0.0001

* Statistical significance of p-values indicates that r differs from 0, but does not reflect the magnitude or clinical relevance of the correlation. AAL: anterior axillary line, CI: confidence interval, MCL: midclavicular line, MSL: midsternal line.
